# Effects of Experienced Discrimination in Pediatric Sickle Cell Disease: Caregiver and Provider Perspectives

**DOI:** 10.1007/s40615-022-01483-4

**Published:** 2022-12-19

**Authors:** Ariel O. Blakey, Claudine Lavarin, Annelise Brochier, Christina M. Amaro, Jenna Sandler Eilenberg, Patricia L. Kavanagh, Arvin Garg, Mari-Lynn Drainoni, Kristin A. Long

**Affiliations:** 1 Department of Psychological and Brain Sciences, Boston University, Boston, MA USA; 2 Department of Health Law, Policy and Management, Boston University School of Public Health, Boston, MA USA; 3 Department of Pediatrics, Boston Medical Center, Boston, MA USA; 4 Department of Behavioral Health and the Center for Healthcare Delivery Science, Nemours Children’s Hospital, Delaware, Wilmington, DE USA; 5 Department of Pediatrics, Boston University School of Medicine, Boston, MA USA; 6 Department of Pediatrics, University of Massachusetts Chan Medical School, Worcester, MA USA; 7 Department of Pediatrics, University of Massachusetts Memorial Health, Worcester, MA USA

**Keywords:** Sickle cell disease, Discrimination, Caregiver perspectives, Pediatrics

## Abstract

For Black children with sickle cell disease (SCD) and their families, high disease stigmatization and pervasive racism increase susceptibility to discrimination in healthcare settings. Childhood experiences of discrimination can result in medical nonadherence, mistrust of healthcare providers, and poorer health outcomes across the lifespan. Caregivers and medical providers are essential to childhood SCD management and are therefore well-positioned to provide insight into discrimination in the context of pediatric SCD. This mixed-methods study sought caregivers’ and providers’ perspectives on processes underlying discrimination and potential solutions to mitigate the negative effects of perceived discrimination among children with SCD. Caregivers (*N* = 27) of children with SCD (≤ 12 years old) and providers from their hematology clinics (*N* = 11) participated in individual semi-structured interviews exploring experiences of discrimination and daily SCD management and completed a quantitative measure of discrimination. Qualitative data were collected until themes reached saturation and subsequently transcribed verbatim, coded, and analyzed using applied thematic analysis. Quantitative and qualitative data converged to suggest the pervasiveness of discrimination in healthcare settings. Three qualitative themes emerged: (1) healthcare system factors underlie discrimination, (2) families’ challenging interactions with providers lead to perceptions of discrimination, and (3) experiences of discrimination impact caregiver-provider interactions. Both caregivers and providers highlighted building trusting patient-provider relationships and encouraging patients’ self-advocacy as means to reduce experiences and impacts of discrimination. These findings offer potential approaches to tangibly mitigate occurrences of discrimination in pediatric healthcare settings by trust building, accountability keeping, and fostering rapport to improve quality of care and pediatric SCD health outcomes.

## Introduction

Sickle cell disease (SCD) is a genetic blood disorder often resulting in multiple complications, resulting in chronic and progressive organ damage, including acute excruciating pain crises, stroke, and life-threatening acute chest syndrome [1]. Black Americans are disproportionately affected by SCD [2]. Sickle cell anemia is a subtype of SCD associated with severe sickle cell–related complications (e.g., severe pain crises) beginning typically in early childhood and often treated by hydroxyurea or penicillin [1].

Structural racism, “the normalization and legitimization of an array of dynamics–historical, cultural, institutional and interpersonal–that routinely advantage White people while producing cumulative and chronic adverse outcomes for people of color,” [4] is a key contributor to experiences of race-based discrimination for Black Americans [3–5]. Given the history of institutionalized racism and perpetuated stereotypes of drug-seeking behavior experienced by Black Americans in medical settings, Black individuals with SCD must often contend with experiences of intersecting disease-based and race-based discrimination [6]. For example, the management of acute, severe SCD-related pain often requires opioid medications, either at home or in the emergency department. However, opioids are associated with biases and stigma towards those who use them, as they are highly addictive and frequently misused in the general population [7, 8], yet this is not often seen in those with SCD [9–11]. Among pediatric populations in the healthcare setting, perceived race-based bias and discrimination are associated with poorer health and mental health outcomes, decreased adherence to medical advice, and increased mistrust in healthcare providers [12–17]. Discrimination encountered during childhood is particularly deleterious for individuals with SCD, due to patient trust and medication/guideline adherence, ultimately affecting the trajectory of their health outcomes across their lifetime [15, 18].

Given the disproportionate prevalence of SCD among Black Americans, stigma attached to individuals who use opioids, and structural racism within medical settings, Black individuals with SCD experience compounding susceptibility to discrimination, beginning in childhood. Yet, the small existing body of research on experiencing intersecting race- and disease-based discrimination in the context of pediatric SCD is limited to adolescents’ self-reports and often exclusively employs quantitative methods [15, 17, 19–21]. Sole reliance on quantitative assessments may underestimate the frequency with which discrimination is reported due to narrow scope of survey questions, which often miss important contextual data of people’s lived experiences and interactions within the healthcare setting [22]. In contrast to previous qualitative SCD literature, which has reported adolescents’ experiences of and reactions to racial bias [17] and highlighted caregivers’ perceptions of racism in the context of inadequate SCD healthcare [23], we integrate the perspectives of both caregivers and providers to explore experienced discrimination particularly in the context of SCD during childhood. Mixed-methods research allows for a multidisciplinary approach [24] and the addition of focused solicitation from multiple respondent perspectives to ensure comprehensive reporting of personal experiences and justification for addressing discrimination. The current study uses a convergent explanatory mixed-methods approach [25] to qualitatively and quantitatively document conceptualizations, experiences, and effects of perceived discrimination in the context of childhood SCD from the perspective of both caregivers and clinical providers.

## Methods

### Participants

Participants were recruited as part of a larger study addressing the implementation of a screening and referral intervention for Social Determinants of Health (SDoH) within the SCD outpatient care setting. For the larger study, primary caregivers participated in qualitative interviews and completed surveys to assess possible mechanisms linking SDoH to SCD outcomes. A purposive sampling approach was used to recruit primary caregivers in person from two hematology clinics to ensure breadth across the child’s gender and age. Eligible caregiver participants were aged > 18 years, spoke English, and identified as the primary caregiver of a child with SCD aged 0–12 years old who was prescribed daily penicillin or hydroxyurea as part of their SCD management. The primary caregiver was defined as the key adult involved in caregiving and SCD disease management (most often the mother of a child with SCD).

In addition, clinical providers (i.e., medical assistants, nurses, physicians, social workers, hereafter referred to as “providers”) from two pediatric hematology clinics involved in the larger SDoH project completed qualitative interviews to examine facilitators and barriers to implementing the SDoH screening and referral intervention. Providers were identified by clinic leadership and were recruited via email. Providers were eligible to participate if they were involved in the clinical care of children with SCD and spoke English. Of the 17 providers contacted, 11 agreed to participate and 6 passively declined by not responding to the email invitation.

### Quantitative Measures

Primary caregivers and providers completed online questionnaires collecting sociodemographic information. Primary caregivers also answered five questions adapted from the Commonwealth 2001 Health Care Quality Survey to assess their experiences of discrimination in the healthcare setting [26] (see Table 1). These yes/no questions asked caregivers if they were ever judged unfairly or treated with disrespect because of their racialized group identity or ethnic background, how well they spoke English, the type of insurance they had, or because their child had sickle cell disease.

**Table 1:** Perceived discrimination questionnaire

Question
1. Was there ever a time when *your child* would have gotten better medical care if *your child* had belonged to a different race or ethnic group?
2. Thinking about all of the experiences *you* have had with healthcare visits in the last 2 years, have you ever felt that the doctor or medical staff you saw judged *you* unfairly or treated *you* with disrespect because: 2a. Of your race or ethnic background? 2b. Of how well you speak English? 2c. Of the type of insurance you have? 2d. Your child has sickle cell disease?

### Qualitative Interviews

Separate semi-structured interview guides were developed for caregivers and providers. The methodological approach to this study was phenomenology, which seeks to understand the phenomenon of perceived discrimination within the sickle cell disease context and its effects. Female authors (AOB, JSE, and CA) conducted caregiver interviews. Female authors (CL and AB) conducted provider interviews. All interviewers represented various professional levels (from graduate students to post-doctoral fellows) and have received relevant training accordingly. Stemming from the larger study, there were some pre-established relationships between some interviewers and the providers. There were no pre-established relationships between the interviewers and the caregivers.

Though specific questions across the two interview guides differed, both were created in alignment with the Health Stigma and Discrimination Framework, which outlines the multilevel consequences of health stigmatization (see Table 2) [27]. Interviews with primary caregivers began with questions to obtain information about the family’s background, social contexts, and experiences with SCD and SDoH. This research focused on questions related to (1) experiences of differential care in healthcare settings; (2) discrimination experienced by families impacted by SCD; and (3) ways caregivers prepared their child with SCD for discriminatory encounters or experiences. Interviews with providers prompted reflection on how public discourse regarding structural racism has impacted clinical teams’ engagement in addressing SDoH, given the interplay between SDoH, health equity, and discrimination. Additionally, providers were asked to describe how their patients reported the effects of bias and discrimination on their healthcare, and to share their thoughts on how to address race- and disease-based bias and discrimination within their clinics, hospitals, and the healthcare system more broadly. Through the course of data collection, new interview questions were added to delve into emerging concepts and others were deemphasized once themes had reached saturation [28].

**Table 2:** Interview guide questions and probes

Caregiver interview guide	Provider interview guide
Lead question 3: Some parents report experiences where they’ve noticed their child has received a difference in their care due to factors they cannot control (i.e., their race or their child’s disease. Please tell me about any experiences you’ve had similar to this 1.How have your encounters with discrimination influenced your experience with doctors, nurses, and other medical professionals who provide care to [child with SCA]? Probes: a.How do you think this discrimination impacted [child with SCA]’s care? b.How did this influence your overall experience and satisfaction with [child with SCA]’s care? c.Within the experiences you described, why do you think you were discriminated against? d.Did medical providers make any assumptions about you based on race, disease presentation, or financial situation? Tell me how e.What could have been improved in this experience? 2.Have there been other healthcare encounters that have been more positive for you? Probes: a.What was different for you in these experiences? 3. What experiences have you heard about from others managing a child with SCA? 4.In what ways have you prepared for discriminatory encounters/experiences?	Lead question: How do you feel the recent conversations about structural racism have impacted provider interest or engagement in addressing unmet basic needs through WE CARE? 1.How do you feel recent events or discussions about structural racism have influenced caregivers’ receptivity to being asked questions about nonclinical needs? To receiving or accessing resources? 2.More broadly how has bias/discrimination affected healthcare in your patient population? Probes: a.On what is the discrimination or bias based? [Race, gender, socioeconomic status, the disease itself, etc.] i.To what extent is it based on sociodemographic characteristics such as race or income versus clinical characteristics? b.What are your thoughts on how to address bias/discrimination within your own clinic? How about in your hospital, or even in the healthcare system as a whole?

### Data Collection

For primary caregivers, semi-structured interviews lasted approximately 60 min and took place via Zoom. All but two primary caregivers gave consent to having their interviews audio-recorded. For two caregivers who declined to record, they provided permission for research staff to take detailed notes. Surveys assessing sociodemographic characteristics and discrimination experiences were administered via REDCap. Primary caregivers were provided $50 for their participation. For providers, semi-structured interviews were audio-recorded and held over Zoom or phone, depending on providers’ preference and availability, and lasted < 60 min. Providers completed sociodemographic surveys via REDCap and were compensated $25 for their participation. Study procedures were approved by the Institutional Review Boards of Boston University Medical Campus Boston University Charles River Campus and Boston Children’s Hospital. Data were collected from March 2020 to March 2021.

### Data Analysis

For the survey data, descriptive statistics were calculated from the sociodemographic survey and quantitative measures using Microsoft Excel 2013. For the qualitative data, recorded interviews were transcribed verbatim, cleaned, and entered in NVivo 12 [29]. Based on a priori research questions and the health stigma and discrimination framework [27], coding structures were developed for the primary caregiver and provider interviews. A subset of transcripts was coded to refine each coding structure based on relevant emerging themes and to ensure reliability among coders (A.O.B., C.L.). The coding structures were deemed final once no new codes were added through this iterative process. All transcripts were coded using the final coding structure. The coding team met weekly to discuss coding and resolve discrepancies through mutual consensus. All coded transcripts were stratified by respondent role (i.e., caregiver vs. provider) and analyzed using applied thematic analysis [30]. Qualitative data were used to give context to the experienced discrimination reported by caregivers in the quantitative survey data.

### Reflexivity

The authors of this manuscript offer a positionality statement to be transparent about our backgrounds and the lenses through which we view research and research processes. Qualitative interviewers were Black and White cisgender young adult and middle-aged females. More broadly, the authors of this manuscript represent various racial/ethnic backgrounds, and religions/spiritual orientation and sexual orientation. Professionally, we hold graduate degrees in Psychology, Education, Public Health, and Medicine. Our academic ranks span master’s and doctoral-level graduate students to Assistant and Associate Professors. Our scholarship uses both qualitative and quantitative methods, and we strive for antidiscrimination in our research and clinical efforts.

## Results

### Sample Description

Interviews were conducted with 27 caregivers and 11 providers. Of the 27 caregiver participants, 25 completed the sociodemographic survey and discrimination measure; all of the providers completed the sociodemographic survey. The majority of caregivers were Black/African American (88.5%), mothers (84.6%), single (53.8%), and publicly insured (57.7%) (Table 3). Providers were all female, and the majority were White (90.9%), non-Hispanic/Latinx (100%), and between 35 and 54 years of age (63.7%). On average, providers had worked in their clinic for approximately 8 years (SD = 6.7), and the majority of participants were nurses (27.3%), hematologists (27.3%), or social workers (27.3%) (Table 4).

**Table 3:** Caregiver and child sociodemographic characteristics (*N* = 26)

	Caregiver	Child
Age, years, mean (SD)	35.7 (8.1)	5.6 (4.2)
Gender, *n* (%) Female Male	23 (88.5) 3 (11.5)	12 (46.2) 14 (53.8)
Country of birth, *n* (%) USA Other Not reported	15 (57.7) 10 (38.5) 1 (3.8)	23 (88.5) 3 (11.5) 0
Race, *n* (%) Black/African American (including Haitian) ^*^ Caucasian or White Other More than one race	24 (92.3) 0 1 (3.8) 1 (3.8)	24 (92.3) 0 1 (3.8) 1 (3.8)
Ethnicity, *n* (%) Hispanic/Latinx	1 (3.8)	1 (3.8)
Health insurance^**,¥^, *n* (%) Public Private None	15 (57.7) 9 (34.6) 2 (7.7)	19 (73.1) 6 (23.1) 1 (3.8)
Medical diagnoses, *n* (%)^¥^ ADHD Asthma Sickle cell disease Cardiac Endocrinologic Hematologic Neurologic Orthopedic Psychiatric	0 3 (11.5) 1 (3.8) 1 (3.8) 2 (7.7) 0 1 (3.8) 1 (3.8) 2 (7.7)	2 (7.7) 2 (7.7) 26 (100) 0 0 1 (3.8) 1 (3.8) 0 1 (3.8)
Caregiver type, *n* (%) Mother Father Grandmother	22 (84.6) 3 (7.7) 1 (43.8)	N/A
Marital status, *n* (%)^¥^ Single Married Separated or divorced	14 (53.8) 8 (30.8) 3 (11.5)	N/A
Highest year of school completed, *n* (%)^¥^ High school graduate or GED Vocational, trade, or business school program Some college credit, but no degree Associate’s degree Bachelor’s degree Master’s degree	2 (7.7) 4 (15.4) 6 (23.1) 4 (15.4) 7 (26.9) 2 (7.7)	N/A

Abbreviations: *ADHD*, attention deficit hyperactivity disorder

^*^One respondent specified their race as “Haitian” when given the “write-in” option

^**^Totals add to more than 100% because of double-insured participants

^¥^Health insurance, medical diagnoses, marital status, and school completed collected for 26 participants

### Quantitative Discrimination Findings

Among caregivers, 40% (*n* = 10) reported experiencing at least one experience of discrimination Among these, six (24%) reported believing that their child would have received better medical care if their child had belonged to a different racialized group identity or ethnic group. Additionally, caregivers reported that doctors or medical staff judged them or treated them with disrespect because of their racialized group identity or ethnic background (20%), how well they spoke English (16%), their child having SCD (24%), and/or their type of insurance (24%). Of the 15 caregivers who did not quantitatively endorse discrimination, nine described experiences of discrimination during the semi-structured interviews (Table 2).

**Table 4:** Practitioner and staff sociodemographics (*n* = 11)

	N (%)
Gender	
Female	11 (100%)
Race	
Black or African American	1 (9.1%)
Caucasian or White	10 (90.9%)
Ethnicity	
Hispanic/Latinx	0
Not Hispanic/Latinx	11 (100%)
Age	
25–34	1 (9.1%)
35–44	3 (27.3%)
45–54	4 (36.4%)
55–64	1 (9.1%)
65–74	2 (18.2%)
Role	
Nurse	3 (27.3%)
Nurse practitioner	1 (9.1%)
Physician	3 (27.3%)
Medical assistant	1 (9.1%)
Social worker	3 (27.3%)
Years worked in clinic	
Mean (SD)	8.0 (6.7)
Median (IQR)	5.0 (12.8)

### Qualitative Discrimination Findings

**Fig. 1:**
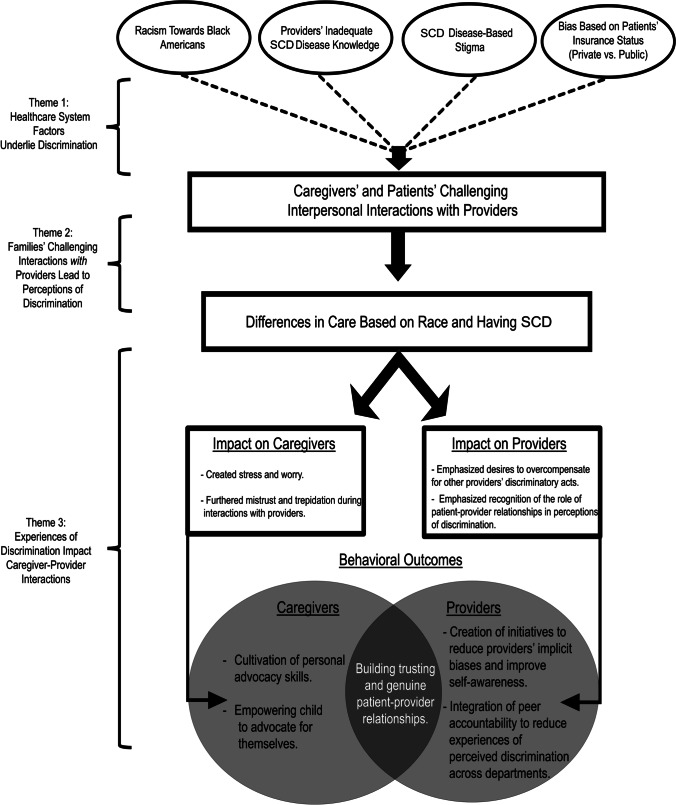
Process and effects of experienced discrimination

Three overarching themes emerged from the qualitative data analysis: (1) healthcare system factors underlie discrimination, (2) families’ challenging interactions with providers lead to perceptions of discrimination, and (3) experiences of discrimination impact caregiver-provider interactions (see Fig. 1).

### Healthcare System Factors Underlie Discrimination (Theme 1)

Caregivers often described racism, inadequate disease knowledge, stigma, and bias as key aspects of the healthcare system that contributed to discriminatory encounters. With regard to racism, caregivers attributed their experiences of discrimination to their own racialized group identity and/or their child’s racialized group identity. Moreover, caregivers believed their likelihood of experiencing discrimination from providers or within the healthcare system broadly would be different if they were White. For example, as one caregiver waited for an appointment for her own child with SCD, she witnessed another Black parent abruptly leave an appointment and say that providers were not giving proper care because the child was Black. Though the caregiver did not have any conversation with that parent, they explicitly reported feeling uncomfortable following this encounter. One White caregiver of an adopted Black child with SCD further expounded upon this phenomenon as she described how her racialized group identity was protective against discriminatory encounters:

"I knew this from when I was learning about sickle cell when I was in nursing school, I don't fit the category of doctors looking down on me, because I am not African American, but I do feel… that doctors might not listen to parents about a child and when I was in [Midwestern State] I certainly felt that– they had not a clue what sickle cell even was. They didn't know what to do with a child like that, and I'm thinking, ‘If I was not White– if I was Black, I don't know…’" (Mother)

Both caregivers and providers reported how providers’ lack of disease knowledge and the “invisible nature” of SCD perpetuate skepticism about the seriousness of symptoms (e.g., pain) and subsequent mistreatment in the healthcare setting. Caregivers and providers both indicated that lack of disease knowledge contributed to providers’ misconceptions about SCD and sustain disease-based stigma.

"I trust [child’s] hematologist and I trust her primary care doctor because they know us and I know they kinda respect us, but sometimes I feel like going into the ER, you have doctors… not as knowledgeable about sickle cell. And I feel like they don't always listen to me when I'm talking and it's not until they talk to her doctor that they, that I feel like they're listening to me." (Mother)

Respondents also emphasized how the intersecting effects of race- and disease-based stigma contributed to families’ increased likelihood of encountering discrimination. While some caregivers solely attributed providers’ assumptions of drug-seeking behavior to disease-based stigma, others emphasized that their Black racialized group identity fed into this assumption. Providers echoed both sentiments, explaining how implicit biases fueled by both historical prejudices towards Black Americans and misperceptions surrounding SCD contributed to discriminatory encounters in the healthcare setting.

"I frequently hear stories about patients feeling discriminated against when they present to the emergency department, how they're looked at as drug seekers based on the amount of pain meds they need, based on the color of their skin, based on the way they're dressed. If they're not feeling well, they're not going to put on a three-piece suit, you know what I'm saying? It really affects a lot of our patients greatly." (Hematology provider)

Lastly, caregivers also reported experiencing discrimination due to bias regarding their insurance status. Caregivers with private insurance reported witnessing and experiencing preferential treatment, while caregivers with public insurance or no access to insurance reported longer wait times and subpar medical care.

"One thing that I do have an issue with is that– a few times that I would go into the emergency room, insurance may have been pending. And I feel like under those circumstances [child] was kind of rushed. [Child] wasn’t properly looked at. And I know when he needs to be admitted… But a few times, [healthcare providers] did a quick little checkup and … sent him home." (Mother)

### Families’ Challenging Interactions with Providers Lead to Perceptions of Discrimination (Theme 2)

Caregivers described examples of challenging interpersonal interactions including being directly spoken to harshly, repeatedly dismissed, or ignored altogether by providers*.* In particular, caregivers emphasized that emergency room providers seemed to overlook their families, ignore their certainty of common SCD-related complications, or prematurely send them home when seeking acute care services. For example, one mother described a provider speaking to her harshly and taking her phone out of her hands to obtain her attention, after having repeatedly ignored her when first bringing her child to the emergency department. She attributed the experience to her being Black, as she observed this provider speaking in a similar “rude way” to another Black patient but speaking in a “different tone” to White patients. Altogether, these interactions often left caregivers feeling disrespected, mistrusting, and concerned that their family received lower-quality healthcare.

"I had asked [child’s providers]– ‘What does this medicine do?… why is my daughter [taking] it?’– and the doctor said, ‘Well, it's not my job to explain to you what it is.’ I said, ‘Oh, oh, but it is…it's your job to explain to me every risk and every pro or con to this…’ I was so angry with them, and I was trying to be nice, but I was livid…I actually felt like [the doctor] felt like, ‘Who does this woman think she is to question me and to make me explain to her and I'm the medical professional?’… like she felt like I'm supposed to just do what she says and that's just it… I felt so disrespected." (Mother)

Caregivers further described experiencing discrimination when they perceived differences in their received healthcare based on racialized group identity or their child having SCD. Experiences of providers’ mistreatment of their children felt unreasonable and left caregivers confused and ultimately concerned about the quality of care afforded to their families.

"When my son is in pain, they keep throwing this behavioral services issue on him. And that is a very upsetting thing, because I can't think of any woman that has gone into labor that has been pleasant. And those are grown adults. So why would you say that… a child that is having bone pain is misbehaving because they're not ‘hello and hi” or even polite about it? Yet, I hear cancer patients, whenever I'm on the floor, screeching and hollering down the hallway, but I don't see security and behavioral services running to…manage their situations. My question is, why would there be behavioral services and security called on a child that can't even walk and has barely enough energy? Why would it take five nurses and four security guards? And this happened twice. And the security guards were standing there and criminalizing him." (Mother)

### Experiences of Discrimination Impact Caregiver-Provider Interactions (Theme 3)

Experiences of discrimination affected how caregivers and providers interacted with one another and engaged with the healthcare system overall. Having to navigate the added responsibility and subsequent stress associated with the uncertainty of how their child would be treated within healthcare settings, in addition to typical parenting and caretaking responsibilities, was a tangible effect of experienced discrimination for caregivers. Caregivers reported being worried and/or scared for their child’s future following their own or others’ experiences of discrimination. However, caregivers also reported that these feelings of fear drove them to increase their disease knowledge and advocacy skills in an attempt to mitigate future encounters with discrimination.

"…it's really sad, because most of sickle cell affects Black people, and I don't know how [providers] see us sometimes. I'm not playing the race card, but… we have to advocate for ourselves. If we don't know, and we just allow them to do whatever they want, we don't get the best treatment. So, we have to advocate for our kids and educate ourselves to know what is out there, that will help the kids. So, [the] communication piece- it’s key.” (Mother)

Many caregivers also expressed the importance of empowering their children to advocate for themselves independently to fortify their abilities to better navigate and overcome potential future encounters of discrimination.

"[Child’s doctor] was very honest and she said, unfortunately, [discrimination] does happen and she's not gonna lie and say that it doesn't. Sometimes [doctors] think people are just there for pain meds, and they don't understand. So [doctor] spoke to the importance of actually going to the hospital where your child received services, and not us just being a support to advocate for [child] but to teach him how to advocate for himself." (Mother)

Providers also described the importance of educating themselves and colleagues to mitigate occurrences of discrimination. In particular, providers reported informally adapting their overarching health systems’ antidiscrimination initiatives for smaller forum settings and encouraging peer accountability across departments to assist in efforts to combat implicit biases and racism contributing to discrimination.

"We've had discussions in our clinic about racism within the healthcare system, how it may be implicit, so we really address that. I think that has definitely made more providers aware of needs. I've always been aware of the sickle cell population having a lot of unmet needs, because they're a very underserved population." (Hematology provider)

Lastly, several providers reported building “trusting relationships” with patients and their families as a way to acknowledge their potential mistrust of providers due to previous experiences of discrimination with other providers and the historically pervasive nature of racism in the general healthcare setting. Providers particularly described feeling the need to be sensitive to patients’/families’ needs and creating safe spaces for conversation.

"… there is a need for us to work even harder in helping these families trust us as the providers, which in large part is a combination of education and experience. It's a bit of both. It's a bit of helping them understand we are here for you." (Hematology provider)

## Discussion

The current research highlights the presence and impact of discrimination within the context of healthcare for children with SCD. Caregivers and providers expressed discrimination, stigma, and racism similarly within healthcare systems. Furthermore, caregivers and providers reported that racism and discrimination contribute to lower-quality care for children across multiple levels of healthcare, which is consistent with previous SCD research [17, 31]. Current findings extend existing research by highlighting how caregivers shifted their behaviors (e.g., by prioritizing the importance of healthy patient-provider rapport and relationships, implementing initiatives to reduce provider biases and increase self-awareness, and holding space for peer accountability across departments) to accommodate the pervasive presence and/or threat of discrimination and bias within healthcare settings.

Differences across respondent types emerged regarding the degree to which discrimination was referenced explicitly. Although caregivers vividly *described* experiences of otherism or differential treatment due to race- or SCD-related stigmas, providers were more likely to *use* the terms racism, discrimination, and stigma. This disconnect between describing and explicitly using the terms racism, discrimination, and/or stigma has been briefly reported in SCD research and may underlie differences between our qualitative and quantitative findings [32]. Similarly, patients with other stigmatizing illnesses, such as substance use disorder and HIV/AIDS, tend not to explicitly name racism as a reason for their differences in care in comparison to their White counterparts [33, 34]. Caregivers’ reasons for describing racism and discrimination yet refraining from explicitly naming those constructs may have important implications regarding how people living with SCD and their families experience, process, and understand discriminatory behavior. Research indicates that there are deleterious biopsychosocial effects of internalized racism in healthcare for patients who are stigmatized [35]. This can manifest in increased levels of stress imposed on the nervous and immune systems, and further employ negative impacts on mental health outcomes [35]. Further research should explore the long-term impacts of discrimination on SCD families, and their engagement with the healthcare system.

Current findings suggest that mistrust between providers and patients may be mitigated by intentional efforts to foster trusting relationships and build rapport between providers and families. Many efforts to address discrimination have been limited to the general field of pediatrics and patient-provider relationships [36–39]. By focusing specifically on pediatric SCD, the current study illuminates the uniquely complex challenges faced by patients with SCD and their families due to combined disease- and race-based discrimination and highlights the need for more robust provider-caregiver-patient approaches across care settings. For example, hematology providers, such as patient advocates, are uniquely positioned to be able to advocate for families of individuals with SCD and improve antidiscriminatory models of treatment between patient families and providers (e.g., trust building, accountability keeping, values, fostering rapport) across healthcare settings [40, 41]. Current findings suggest the need for communal gatekeeping of self and team accountability within larger healthcare provider networks to mitigate discriminatory effects on patients and families as they seek care in the emergency department or inpatient setting. Existing models may be adapted for the SCD population. Peer accountability and self-awareness may further serve as mechanisms to mitigate stigma, bias, and discrimination in healthcare [42], but additional approaches are needed to address the intersection between race- and disease-related stigma and bias. Systemic and institutional approaches may be best to reduce discrimination to shift the responsibility for seeking antidiscriminatory solutions to providers and away from families.

Our findings can be understood in the context of historic and current racism broadly within the US and specifically within the healthcare system. For example, healthcare providers’ perceptions of, and influence on, Black individuals with SCD may be affected by the broader history of pain medication overuse and abuse in the USA and aspects of structural racism, which together may amplify the persistent drug-seeking stigma among Black individuals with SCD [6, 8, 16, 17, 43]. The pervasive nature of discrimination and differential treatment has been well documented within the healthcare system broadly [44], and for SCD within the emergency department [43, 45, 46] and inpatient settings [47, 48], including challenging interpersonal interactions with medical providers. The current work also highlights discrimination related to insurance, which is not yet represented in available literature despite high rates of Medicaid usage among pediatric SCD populations [49].

Lack of awareness of SCD in the healthcare system may contribute to the invisibility of the disease itself [50]. SCD is often referred to as an invisible disease for various reasons, such as the difficulty of objectively identifying the characteristics and symptoms, the lack of physical or laboratory markers of pain, and the systemic marginalization of the people that it predominantly affects [51]. In addition, there is less funding, research, and advancements for SCD compared to similar diseases that predominantly affect White Americans [3, 52]. For example, cystic fibrosis (CF) has three times more federally approved medications/treatments and receives up to 11 times more funding than SCD [3, 52]. Yet, CF impacts less than one-third as many people as SCD (100,000 to 30,000) [3, 52]. Increasing awareness and education regarding SCD may equip providers to more effectively care for children and adults with SCD and may help to expand funding for research to inform efforts to mitigate the presence and negative effects of discrimination within healthcare settings. Drawing upon findings from other stigmatized illnesses such as HIV/AIDS and substance use disorder, promising approaches to mitigate discrimination include ensuring appropriate descriptive language and naming of stigmatized populations within healthcare settings, listening to patient stories, and including and encouraging familial and/or community support [53, 54].

Current findings should be considered in light of limitations. First, the sample of two clinics and 27 caregivers from the Northeast region of the USA may not be generalizable to the broader SCD population. Second, providers were all female and nearly all non-Latinx White. This may not be representative of many hematology clinics. Third, caregivers of children between the ages 0–12 years old may have different experiences than caregivers of adolescents. Yet, these early experiences of children and caregivers are important to capture as they may have implications for their life course. Though the nature of agency and autonomy of their healthcare engagement may change adolescence and young adulthood, at which point caregivers’ awareness of discrimination is subject to change. Additionally, the measure used to quantitatively evaluate discrimination was adapted from an existing health quality survey [25] and as such is not an independently validated measure of discrimination. Future research should seek to utilize a validated measure of discrimination to improve the replicability of reliable and accurate results. Lastly, there may be bias or missed information present among participants who decided not to participate versus those who agreed to participate in the study. Additional funding for research, community partnership, and engagement between healthcare institutions and SCD populations may help to improve the quality of care for people living with SCD [42].

In conclusion, this research highlights the experiences of discrimination, stigma, and bias of families of children with SCD within the healthcare system. By drawing on the perspectives of both caregivers and providers, these findings suggest promising approaches to reduce the frequency of discrimination in healthcare, foster healthier relationships and partnerships between patients/families and providers, and improve the quality of care across the healthcare system. Together, this may lead to improved healthcare experiences and trajectories of health outcomes for individuals with SCD across the life course.

## Data Availability

The datasets used and/or analyzed during the current study are available from the corresponding author on reasonable request.
